# Emergence of SARS-COV-2 Spike Protein Escape Mutation Q493R after Treatment for COVID-19

**DOI:** 10.3201/eid2710.211538

**Published:** 2021-10

**Authors:** Daniele Focosi, Federica Novazzi, Angelo Genoni, Francesco Dentali, Daniela Dalla Gasperina, Andreina Baj, Fabrizio Maggi

**Affiliations:** Pisa University Hospital, Pisa, Italy (D. Focosi);; Azienda Socio-Sanitaria Territoriale, Varese, Italy (F. Novazzi, F. Dentali, A. D. Dalla Gasperina, A. Baj, F. Maggi);; University of Insubria, Varese, (A. Genoni, F. Dentali, D. Dalla Gasperina, A. Baj, F. Maggi)

**Keywords:** severe acute respiratory syndrome coronavirus 2, SARS-CoV-2, coronaviruses, viruses, coronavirus disease, COVID-19, respiratory infections, spike protein, spike protein escape mutation Q493R, variant, receptor-binding motif, treatment, bamlaniv/etesevimab, zoonoses, Italy

## Abstract

We report in vivo selection of a severe acute respiratory syndrome coronavirus 2 spike mutation (Q493R) conferring simultaneous resistance to bamlanivimab and etesivimab. This mutation was isolated from a patient who had coronavirus disease and was treated with these drugs.

Variants of severe acute respiratory syndrome coronavirus 2 (SARS-CoV-2) usually result from random mutations in humans or other hosts, but accelerated evolution can also occur under selective pressure from therapeutic interventions using neutralizing antibodies (*1*). Bamlanivimab has been recently withdrawn as a monotherapy because of treatment failure against E484K SARS-CoV-2 virus variants. Emergency use remains authorized for the bamlanivimab/etesevimab cocktail, targeting overlapping epitopes (*2*), for which no completely resistant variant has been reported to date. This cocktail has been effective in reducing hospitalizations when administered early after infection (*3*).

Given increasing reports of accelerated intrahost evolution of drug-resistant SARS-CoV-2 clades after neutralizing antibody‒based treatments (*4*,*5*), we began screening patients who failed to show virus-negative results for nasopharyngeal swab (NPS) specimens after they were given bamlanivimab/etesevimab. We report an in vivo case of a spike protein escape mutation conferring combined resistance to bamlanivimab and etesevimab.

A 73-year-old man had cholangiocarcinoma diagnosed during February 2021. While he was waiting for chemotherapy, sepsis developed, and he was admitted to Varese Hospital (Varese, Italy) on April 12 and given a steroid and antimicrobial drugs. At admission, reverse transcription PCR (RT-PCR) for SARS CoV-2 in an NPS specimen showed a negative result, but the same test showed a positive result on April 24. 

Given that he had recovered from sepsis, the patient was moved to the coronavirus disease unit of the hospital on April 25. He satisfied 1 of the frail-patient categories for emergency use of spike protein monoclonal antibodies approved by the Italian Drug Agency. The patient was also seronegative for S1/S2 IgG against spike protein (Diasorin, https://www.diasorin.com). 

On April 26, the patient received a single intravenous infusion of bamlanivimab (700 mg) and etesevimab (400 mg) at the hospital. RT-PCR performed on an NPS specimen collected before the infusion was positive for SARS-CoV-2 and showed a cycle threshold (C_t_) of 12 (Alinity Analyzer; Abbott Laboratories, https://www abbott.com).

Follow-up analysis of NPS specimens showed positive results on Apr 28 (C_t_ 15) and May 3 (C_t_ 24). Chest computed tomography on April 30 showed progression to interstitial pneumonia, and the patient was given noninvasive ventilation. No additional bamlanivimab/etesevimab infusion was performed, and the patient died on May 14.

According to national guidelines for breakthrough infections, we sequenced SARS-CoV-2‒positive samples. We performed a SARS CoV-2 RT-PCR on NPS specimens by using the Alinity Platform (Abbott Laboratories), and measured S1/S2 IgG by using a chemiluminescent immunoassay (Diasorin). We used the Sanger method to sequence the spike gene as reported (*6*), analyzed sequences by using NextStrain (https://nextstrain.org), and deposited sequences in GenBank.

Spike gene sequencing of the NPS specimen obtained on April 24 clade B.1.1.7 (Alpha; NextStrain clade 20I/501Y.V1; GenBank accession no. MZ157261), which was 94% prevalent in Italy at that time. However, the May 3 specimen showed a secondary A1478G peak in the spike protein gene, corresponding to the spike Q493R mutation, which became predominant by May 8 (C_t_ 18; GenBank accession no. MZ157275) (Figure).

**Figure:**
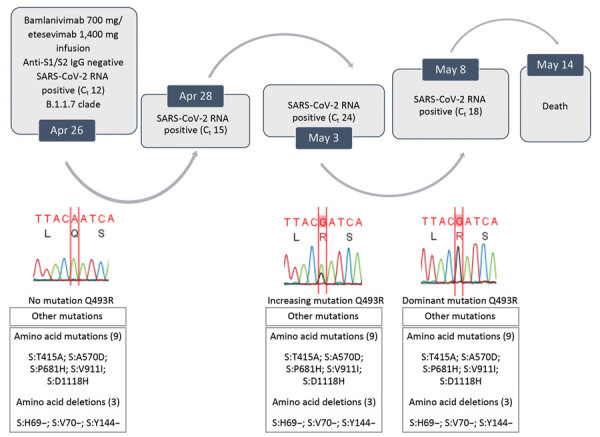
Evolution of SARS-CoV-2 variants in a patient who had coronavirus disease who was given bamlanivimab and etesivimab, Italy. C_t_, cycle threshold; SARS-CoV-2, severe acute respiratory syndrome coronavirus 2.

E484, F490, Q493, and S494 are the 4 aa residues within the spike protein receptor-binding motif that are known to be critical for bamlanivimab binding. Q493 is also among the many more receptor-binding motif residues crucial for interactions with etesivimab. Q493R/K (which can be selected in vitro by bamlanivimab [*7**,**8*]) is to date the only mutation that causes resistance to bamlanivimab and etesivimab. This residue also causes resistance to other class 3 monoclonal antibodies (*8*) (i.e., those that do not overlap with the a*ngiotensin-converting enzyme 2* binding site and have accessibility to the receptor-binding domain epitope in the up and down conformations).

In pseudoviral neutralization assays, Q493R reduces susceptibility to bamlanivimab by >6,666-fold, to etesevimab by 232-fold, and to the combination of both drugs by >100-fold (*2*). In a flow cytometry competitive assay, Q493R reduces the 50% inhibitory concentration >100-fold for bamlanivimab and 42-fold for etesivimab (*7*). Q493R has a frequency of 0.006% in the GISAID database (https://www.gisaid.org; 85 of 1,424,998 deposited sequences as of May 8, 2021; https://covid19dashboard.regeneron.com/?tab=Mutation_Details&subTab=Spike), making the occurrence of co-infection with a Q493R-positive strain extremely unlikely in our patient. 

It remains unclear how such risk extends to different spike protein monoclonal antibody cocktails targeting nonoverlapping epitopes. Although different mutations can similarly cause immune escape by the nonoverlapping REGN-CoV-2 (imdevimab plus casirivimab) cocktail, hamster models and clinical trials showed no increased emergence of variants (R. Copin et al., Regeneron Pharamaceuticals Inc., pers. comm., 2021 Jun 22). Nevertheless, Choi et al. reported a patient having detectable SARS-CoV-2 for 154 days, and accelerated viral evolution in the spike protein after being given remdesivir and REGN-CoV-2 (*4*).

The nonoverlapping AZD7442 (COV2–2130 and COV2–2196) cocktail also seems resistant to rapid escape (J. Dong et al., Huazhong University of Science and Technology, pers. comm., 2021 Jun 21), but again, such in vitro or animal models could miss rare in vivo events. 

In conclusion, SARS-CoV-2 mutations conferring resistance to bamlanivimab and etesevimab can arise in vivo after specific selective pressure; Q493 mutations increase binding affinity to the a*ngiotensin-converting enzyme 2. Additional* studies are needed to clarify whether such escape mutations can spread and persist in humans. Genomic surveillance for SARS-CoV-2 variants is encouraged for coronavirus disease patients who do not respond well to treatment with spike protein monoclonal antibodies.
